# Dengue Fever With Fulminant Liver Failure and Fatal Pulmonary Alveolar Hemorrhage: A Case Report

**DOI:** 10.7759/cureus.28302

**Published:** 2022-08-23

**Authors:** Asheesh Gautam, Harshpreet Singh

**Affiliations:** 1 Internal Medicine, Government Medical College & Hospital, Chandigarh, Chandigarh, IND

**Keywords:** acute dengue, acute viral hepatitis, fluid-refractory shock, diffuse alveolar hemorrhage, n- acetyl cysteine, acute fulminant liver failure, dengue fever/complications

## Abstract

Dengue infection may rarely present with end-organ dysfunction. A 22-year-old male patient presented with a serologically confirmed dengue infection, with clinical manifestations and laboratory pictures suggestive of fulminant hepatitis. The in-hospital disease course was complicated with encephalopathy, recurrent hypoglycemic episodes, coagulopathy, pulmonary alveolar hemorrhage, hypotension, and kidney injury. He was managed with intravenous fresh frozen plasma, platelet concentrate, crystalloids and N-acetyl cysteine (NAC) along with other recommended supportive measures for dengue and fulminant hepatic failure. The patient did not show any improvement in liver function despite therapy and succumbed to his illness on day 6 of hospitalization. In view of the large burden of disease in developing nations and atypical manifestations of dengue infection, research into effective treatment strategies is warranted.

## Introduction

Dengue fever (DF) is a common arboviral infection transmitted by* Aedes aegypti.* Developing nations face a high disease burden especially in the monsoon season due to vector multiplication in water-logged places. The disease has a myriad of clinical presentations such as asymptomatic illness, febrile illness, dengue haemorrhagic fever (DHF), or dengue shock syndrome (DSS). Liver failure or hepatitis, encephalitis, acute kidney injury, transverse myelitis, and myositis are a few under-reported manifestations of dengue infection. Hepatic involvement in dengue can range from the commonly seen mild elevation of liver enzymes to rare acute fulminant liver failure [1]. Intravenous hydration, packed red blood cells (pRBC) transfusion to improve oxygen delivery to hepatic cells, and the use of N-acetyl cysteine (NAC) are some common treatment modalities with unknown efficacy [2]. Pulmonary involvement in dengue, especially pulmonary alveolar haemorrhage, is rare or is under-reported [3]. Presentation of dengue infection as acute fulminant liver failure with pulmonary haemorrhage is unusual and carries a high rate of mortality.

## Case presentation

A 22-year-old male resident of Rupnagar, Punjab presented to the emergency department with a history of high-grade fever with chills for three days associated with episodes of vomiting, abdominal pain, yellowish discoloration of the skin, and progressive deterioration in sensorium with confusion, restlessness, and agitated behaviour. No history of consumption of ethanol, painkillers, or antipyretics beyond prescription dose was present.

**Figure 1:**
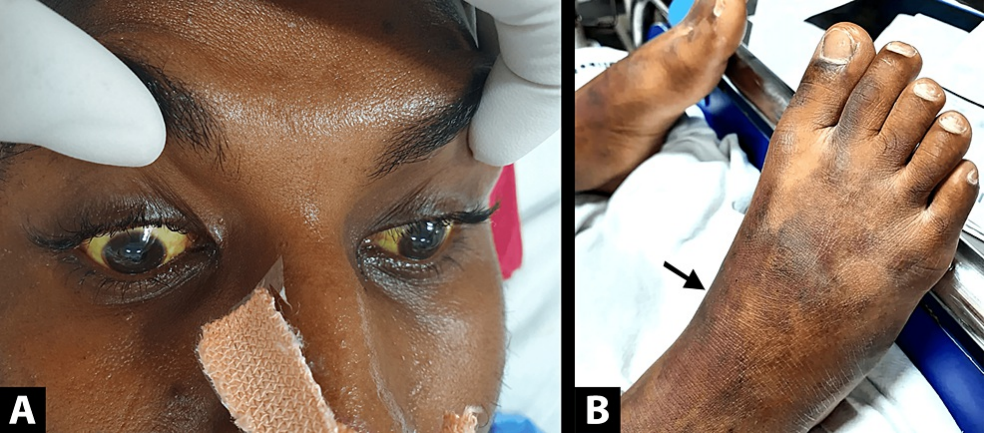
A) icteric sclera and skin, B) confluent ecchymotic areas over anterior aspect of bilateral legs and dorsum of feet

On examination, the patient was drowsy and not oriented to time, place, or person. Bilateral pupils were reactive to light and bilateral plantars showed flexor response. There were no features of focal neurological deficit or meningeal irritation. Icterus involving sclera and skin (as seen in Figure 1A) and ecchymotic patches over the anterior aspect of bilateral legs were present (Figure 1B). The patient was hypotensive (mean arterial pressure (MAP): 60 mmHg), tachycardic (PR: 118/minute), and had respiratory distress (respiratory rate: 32/min, room air oxygen saturation: 68%) on presentation. On chest auscultation, normal vesicular breath sounds were present without any added sounds. Abdomen was soft with no obvious hepatosplenomegaly. He was managed during the initial six hours with fluid therapy with no increase in MAP, followed by inotropic support.

**Figure 2:**
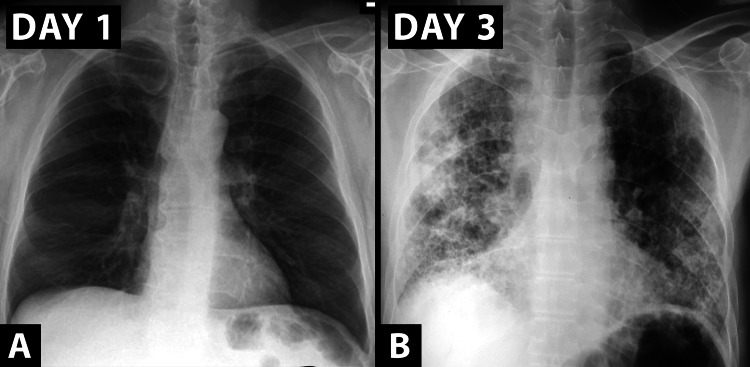
Chest radiographs showing A) normal findings at presentation, B) development of bilateral heterogenous opacities with peripheral predominance, likely alveolar hemorrhage

**Figure 3:**
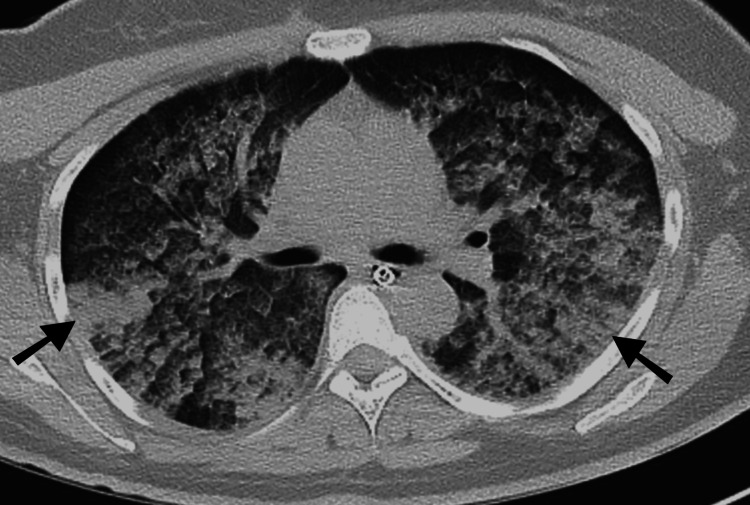
High-resolution CT (HRCT) chest done on day 4 showing ground-glass opacities in bilateral lung fields

In view of the failure to respond to the titrated dose of noradrenaline, persistent hypoxia, and declining sensorium, an endotracheal intubation was done and mechanical ventilation was started. Broad spectrum antibiotics were started and intravenous NAC was given for acute liver failure. Prolonged hypotension led to acute renal injury and the patient remained anuric throughout the hospital course. Blood pressure improved after 12 hours of standard fluid therapy and inotropic support but thrombocytopenia and coagulopathy manifested as pulmonary hemorrhage with visible endotracheal bleed and oronasal bleed on day 2. Recurrent episodes of hypoglycemia were observed and the patient was placed on continuous dextrose infusion during his hospital stay. Initial chest radiograph was normal (Figure 2). Plain CT scan of the brain did not show any intracranial pathology. Abdominal ultrasonogram revealed mild ascites, raised periportal echogenicity, and hepatomegaly (liver size: 16.5 cm). Serum tested positive for dengue IgM and IgG antibodies. Other infectious causes of shock and acute liver failure were ruled out (negative results for scrub typhus IgM, leptospira IgM, malarial antigen, and hepatitis A, E, B, and C). With treatment, lactate levels improved but liver dysfunction persisted and renal dysfunction worsened. Hemoglobin levels (after the initial drop) and platelet count gradually improved with blood product transfusions (Table 1). Repeat chest radiograph showed diffuse opacities (as seen in Figure 2B) and high-resolution CT (HRCT) chest showed multiple regions in bilateral lung fields with ground-glass opacities, suggestive of pulmonary alveolar haemorrhage (Figure 3). Inotropic support was gradually tapered and stopped on day 4 of hospitalization and the patient stayed normotensive on intravenous fluid support.

**Table 1:** Investigation trends showing persistent hepatic dysfunction and worsening renal function. Hemoglobin and platelet levels improved after multiple blood product transfusions Hb: hemoglobin, INR: international normalized ratio, TSB: total serum bilirubin, CSB: conjugated serum bilirubin, AST: aspartate transaminase, ALT: alanine transaminase.

Day of hospitalization	1	2	3	4	5	6
Hb (gm/dL)	7.5	3.8	5.9	6.6	7.7	7.2
Platelets (*10^9^/L)	14	29	30	46	62	60
INR	2.35	1.97	1.74	2.05	1.50	2.10
Lactate (mmol/L)	7.2	6.2	6.2	2.6	2.2	2.9
Urea (mg/dL)	170	190	255	228	254	271
Creatinine (mg/dL)	3.6	4.1	4.5	4.5	7.2	7.4
TSB (mg/dL)	4.8		6.3		7.4	
CSB (mg/dL)	2.6		5.9		6.3	
AST (IU/L)	12040		10392		11320	
ALT (IU/L)	4230		5120		8071	

Injectable vitamin K, tranexamic acid, and etamsylate were also prescribed in an attempt to cease ongoing bleeding status, with no effect, and the patient continued to bleed. In total, four pRBCs, two platelet concentrates, four platelet-rich plasma, and 12 fresh frozen plasma units were transfused. Ventilator support requirements progressively increased. Despite exhaustive medical management, the patient could not be saved and succumbed to his illness on day 6 of hospitalization due to respiratory failure.

## Discussion

Acute liver failure caused by DF is remarkably rare; retrospective studies have reported a prevalence range of 0.3% - 1.1% [4]. Although elevated aspartate transaminase (AST) and alanine transaminase (ALT) levels are seen in 63%-97% and 45%-96% of dengue patients, only 4% of cases have a 10-fold transaminitis increase [5]. Postulated pathogenic hypotheses includes direct viral effect causing hepatocyte necrosis and apoptosis, cell injury due to host immunity, circulatory compromise, metabolic acidosis, and hypoxia caused by hypotension or localized vascular leakage inside the liver [6]. Evidence-based guidelines regarding the management of acute liver failure in dengue are few. NAC is an antioxidant agent which is known to restore the hepatic glutathione reserve used in acetaminophen-related liver injury. However, the use of NAC in other causes of liver failure, particularly in DF-related liver injury, has gained importance in the recent past [2]. Benefit has been observed when NAC is used in the early stages of liver failure but not in the advanced stages [7].

Pulmonary hemorrhage is a rare complication of DHF, seen in 1.4% of cases. Although pulmonary function may rarely improve after cessation of bleeding, it carries a high rate of mortality in most cases [3].

Effective management in complicated dengue infection is elusive, with no definitive treatment and only supportive care being recommended.

## Conclusions

Dengue infection causing acute fulminant liver failure with progression to multiple organ dysfunction was observed in this patient. Hepatic dysfunction was not responsive to medical therapy and led to pulmonary alveolar hemorrhage. This is an atypical presentation of DF and carries a high rate of mortality due to the lack of effective treatment strategies. Given the large global burden of the disease, research concerning definitive therapy and management guidelines is needed.
